# Establishing Institutional Scores With the Rigor and Transparency Index: Large-scale Analysis of Scientific Reporting Quality

**DOI:** 10.2196/37324

**Published:** 2022-06-27

**Authors:** Joe Menke, Peter Eckmann, Ibrahim Burak Ozyurt, Martijn Roelandse, Nathan Anderson, Jeffrey Grethe, Anthony Gamst, Anita Bandrowski

**Affiliations:** 1 Center for Research in Biological Systems University of California, San Diego La Jolla, CA United States; 2 SciCrunch Inc. San Diego, CA United States; 3 Department of Neuroscience University of California, San Diego La Jolla, CA United States; 4 Martijnroelandse.dev Ouderkerk aan de Amstel Netherlands; 5 Department of Mathematics University of California San Diego, CA United States

**Keywords:** research reproducibility, rigor and transparency, reproducibility crisis, reporting transparency, science of science, research metric, data and code availability, cell line authentication, university ranking

## Abstract

**Background:**

Improving rigor and transparency measures should lead to improvements in reproducibility across the scientific literature; however, the assessment of measures of transparency tends to be very difficult if performed manually.

**Objective:**

This study addresses the enhancement of the Rigor and Transparency Index (RTI, version 2.0), which attempts to automatically assess the rigor and transparency of journals, institutions, and countries using manuscripts scored on criteria found in reproducibility guidelines (eg, Materials Design, Analysis, and Reporting checklist criteria).

**Methods:**

The RTI tracks 27 entity types using natural language processing techniques such as Bidirectional Long Short-term Memory Conditional Random Field–based models and regular expressions; this allowed us to assess over 2 million papers accessed through PubMed Central.

**Results:**

Between 1997 and 2020 (where data were readily available in our data set), rigor and transparency measures showed general improvement (RTI 2.29 to 4.13), suggesting that authors are taking the need for improved reporting seriously. The top-scoring journals in 2020 were the *Journal of Neurochemistry* (6.23), *British Journal of Pharmacology* (6.07), and *Nature Neuroscience* (5.93). We extracted the institution and country of origin from the author affiliations to expand our analysis beyond journals. Among institutions publishing >1000 papers in 2020 (in the PubMed Central open access set), Capital Medical University (4.75), Yonsei University (4.58), and University of Copenhagen (4.53) were the top performers in terms of RTI. In country-level performance, we found that Ethiopia and Norway consistently topped the RTI charts of countries with 100 or more papers per year. In addition, we tested our assumption that the RTI may serve as a reliable proxy for scientific replicability (ie, a high RTI represents papers containing sufficient information for replication efforts). Using work by the Reproducibility Project: Cancer Biology, we determined that replication papers (RTI 7.61, SD 0.78) scored significantly higher (*P*<.001) than the original papers (RTI 3.39, SD 1.12), which according to the project required additional information from authors to begin replication efforts.

**Conclusions:**

These results align with our view that RTI may serve as a reliable proxy for scientific replicability. Unfortunately, RTI measures for journals, institutions, and countries fall short of the replicated paper average. If we consider the RTI of these replication studies as a target for future manuscripts, more work will be needed to ensure that the average manuscript contains sufficient information for replication attempts.

## Introduction

### Background

Research reproducibility is necessary for scientific progress. However, over the last decade, numerous reports on research irreproducibility have shed light on a lingering problem, one that is proving to be both troublesome and costly [1-5]. Ioannidis [1] and the Open Science Psychology collaboration examined the issue from a statistical point of view, arguing that multiple comparisons that are not necessarily reported affect the published literature. Begley and Ellis [2] described an account in which their teams attempted to reproduce key cancer studies and were largely unable to do so; however, they did not share their data. The Center for Open Science recently published a series of papers summarized by Errington et al [6], which describe an open replication attempt that had similar findings to the work by Begely and Ellis [2]. Vasilevsky et al [4] clearly showed that approximately half of the reagents in papers cannot be tracked down, whereas Freedman et al [7] attempted to visualize the economic impact of irreproducibility.

Fortunately, many stakeholders responded to address these issues. Funders such as the National Institutes of Health (NIH), the largest public source of health research funding worldwide [8], have made significant efforts across multiple fronts. The NIH advanced open publication efforts with the creation of PubMed Central. In terms of guidelines, the NIH gathered copious stakeholder feedback and designed and implemented rigor and reproducibility guidelines (adapted from the study by Landis et al [9]). The NIH also rewrote their instructions to grantees, released numerous training modules and webinars, and implemented a data sharing policy to improve the reproducibility of funded research [10,11]. Even some private funders such as the Gates Foundation have begun requiring their funded research (both the manuscript and its data) to immediately become open access once published [7].

Journals and publishers have also responded to this. In an effort to encourage reproducibility, numerous scientific organizations and journals have adopted the Transparency and Openness Promotion guidelines, which focus on establishing best practices at the level of individual journals [12]. Similarly, the publisher-driven Materials Design, Analysis, and Reporting framework is a multidisciplinary research framework designed to improve reporting transparency across life science research at the level of individual manuscripts [13]. This framework provides a consistent, minimum reporting checklist whose criteria were used in part to create the first Rigor and Transparency Index (RTI), a journal quality metric focusing on research methodologies and transparency in reporting [14]. Because of the RTI, journals can be compared using a range of criteria that impact reproducibility, providing a proxy for research quality and a strong incentive for improvement.

Unfortunately, these types of indicators and incentives do not exist for all stakeholders. Research institutions, in particular, have few options for determining whether investigators will follow the guidelines. In fact, there is no simple way to see a university’s corpus, let alone to estimate its quality. Despite previously contributing to the *Reproducibility Crisis* [15], institutional output is still difficult to track and measure. Various systems for ranking faculty are in place at institutions, including counting publications, counting citations, and counting *high impact* publications; however, issues have been reported when using the impact factor for these purposes [16,17]. Some institutions started leaning more heavily on assessments of open science [18], which reduced the reliance on paper counting or on the impact of particular journals. Indeed, tying researcher assessment to any single factor, even if that happens implicitly by reviewers looking for recognizable journal names, may place inappropriate pressure on scientists to focus on strategies that increase research notoriety rather than quality, which can have wider implications [19,20].

After receiving feedback from several stakeholders [21,22], we developed a new version of SciScore, an automated natural language processing tool suite that detects transparency criteria and research resources within individual papers. In conjunction with this, we linked published manuscripts with their disambiguated research institutions. Here, we introduce the latest version of the RTI, version 2.0, which represents the mean SciScore over a subset of papers and demonstrates how it can be used to assess reporting transparency within research institutions. The fact that the MacLeod laboratory is endeavoring to register a report assessing institutions on similar metrics (MacLeod personal communication) suggests the importance of assessing based on quality rather than citations alone.

### Objectives

The overall aim of this study was to establish a scientific reporting quality metric across institutions and countries and to highlight the need for high-quality reporting to ensure replicability within biomedicine, using manuscripts from the PubMed Central Open Access Initiative and the Reproducibility Project: Cancer Biology [6].

## Methods

### Individual Manuscript Processing

#### Overview

Individual manuscripts were processed using the latest version of SciScore (research resource identifier [RRID]: SCR_016251). SciScore uses multiple conditional random field (CRF)-based models [23] in combination with regular expression patterns for named entity recognition. For more information on the core features used within CRF models, please see our previous work on the Resource Disambiguator for the Web, which used the same framework [24]. SciScore classifiers currently recognize 27 entity types. New entity types include field sample permits, general euthanasia statements, inclusion and exclusion criteria, attrition, general replication statements, number of replications, type of replication, age, weight, code availability, data availability, and statistical tests. Table S1 in Multimedia Appendix 1 provides a full list of entity types and their descriptions.

Classifiers were validated using precision, recall, and their harmonic mean (*F*_1_). Their initial performances were calculated using 10 random splits of the human-curated data, where 90% was used for training and 10% for testing; each performance score was the average of all 10 training trials. Classifier performances are listed by entity type in Table S2 in Multimedia Appendix 2. The study by Menke et al [14] provides the full description of how the data sets were labeled and how the classifiers were trained and tested. In addition to its CRF-based classifiers, SciScore has begun to implement regular expressions for detecting protocols, data, and code identifiers. Regular expression pattern sets were initially adapted from the identifier patterns listed by [25] (RRID: SCR_003735). These sets were then adjusted and supplemented accordingly. These patterns are listed in Multimedia Appendix 3.

In addition, enhanced table detection and tabular data extraction within SciScore were performed using neural network models. More specifically, table and section header boundary detection and subsequent table row detection in the provided free text were performed with feedforward neural networks using a sliding context window approach.

##### New Criteria and Scoring Framework

Of the new criteria added (ie, field sample permits, general euthanasia statements, euthanasia agents, inclusion and exclusion criteria, attrition, general replication statements, number of replications, type of replication, age, weight, protocol identifiers, code availability, code identifiers, data availability, data identifiers, and statistical tests), the vast majority have been implemented in RTI, version 2.0. When creating the manually checked data sets, we grouped euthanasia and euthanasia agents to align with the output of the automated pipeline. Some criteria presented in SciScore’s output, namely oligonucleotides and statistical tests, were also omitted in terms of scoring, where we continued to refine their natural language processing algorithms.

The scoring framework was previously described in our study using RTI, version 1.0 [14]. To summarize the key findings, research papers were scored on a 10-point scale, where a maximum of 5 points was derived from the manuscript’s rigor adherence and another 5 points from its key resource identification performance. A comparison of the total number of identified criteria with the total number of expected criteria provided the rigor adherence score. Please note that currently, code availability, data availability, and the various identifiers (protocol, code, and data) do not yet affect scoring (ie, they do not contribute to found or expected tallies). This will be addressed in future studies.

Following a similar found-to-expected scoring system, key resource identification performance is calculated by comparing the number of uniquely identifiable resources found (ie, those with RRIDs or RRID suggestions) to the total number of resources detected. If no resources or criteria were found or if the only criteria found does not impact scoring (code availability, data availability, protocol identifiers, code identifiers, data identifiers, statistical tests, and oligonucleotides), then the paper was scored as a 0 and was considered *not applicable*. Papers with a score of 0 were excluded from the data set because there was no way to determine if scoring was appropriate.

Other than the addition of new criteria, the only key scoring change between RTI, version 1.0, and RTI, version 2.0, was the inclusion of more conditional scoring logic within the rigor adherence section. In RTI, version 1.0, the only conditional scoring logic being implemented involved cell line authentication, which was only expected when a cell line was detected in the manuscript. In RTI, version 2.0, an additional scoring logic was included. This logic is outlined in Table 1. As an example, if a criterion was found in the ethics-1 grouping (IACUC, IRB, or consent), the model would expect at least one of the group selection criteria (inclusion and exclusion criteria or attrition), sex, at least one of the demographic criteria (age or weight), randomization, blinding, and power analysis. If a manuscript contained an IACUC and age but no other criteria, the model would detect 2 out of 6 expected criteria, which translates roughly to a 2 out of a maximum 5 points for this section. As another example, if euthanasia was detected, we would expect Institutional Animal Care and Use Committee, Institutional Review Board, or consent.

**Table 1:** Conditional scoring groupings and logic for rigor adherence section.

Grouping	Criteria included	If this grouping is detected, what is expected?	This grouping is expected when what is detected?
Ethics-1	Institutional Animal Care and Use Committee, Institutional Review Board, and consent	Group selection, sex, demographics, random, blinding, and power	Euthanasia
Ethics-2	Field sample permit	Random, blinding, and power	Never expected
Euthanasia	Euthanasia statement and euthanasia agent	Ethics-1, group selection, sex, demographics, random, blinding, and power	Never expected
Group selection	Inclusion and exclusion criteria and attrition	Random, blinding, and power	Ethics-1 and euthanasia
Sex	Sex	Random, blinding, and power	Ethics-1, euthanasia, and demographics
Demographics	Age and weight	Sex, random, blinding, and power	Ethics-1 and euthanasia
Random	Random	Blinding and power	Always expected
Blinding	Blinding	Random and power	Always expected
Power	Power analysis	Random and blinding	Always expected
Replication	Replication statement, number of replications, and type of replication	Random, blinding, and power	Never expected
Cell line authentication	Cell line authentication and cell line contamination	Sex, random, blinding, and power	Cell lines
Methods and materials availability^a^	Data availability, data identifiers, code availability, code identifiers, and protocol identifiers	Never expected; do not affect score	Never expected; do not affect score
Cell lines	Cell lines	Cell line authentication	Never expected
Other resources^b^	Antibodies, organisms, plasmids, and tools	Never expected; only affects resource transparency score	Never expected; only affects resource transparency score
Miscellaneous^a^	Oligonucleotides, statistical tests, and incorrect research resource identifiers	Never expected; does not affect either score	Never expected; does not affect either score

^a^Row indicates criteria that do not affect any score.

^b^Row indicates criteria that do not affect the rigor adherence score, only the resource transparency score.

##### Validation

Although some entity types have been previously tested (cell lines in the study by Babic et al [26] and multiple types in the study by Menke et al [14]), other entity types and regular expression patterns have not yet been thoroughly validated on complete articles outside of training sets. To remedy this, we tested the performance of our models using 423 papers that were previously selected at random for manual curation during testing using RTI, version 1.0. Originally, 2 sets of 250 papers were randomly selected based on their score during the first run in November 2019 (SciScore>0: 250 papers; SciScore=0: 250 papers). We used these hand-curated papers as the gold standard to retest performance during testing by RTI, version 2.0, to ensure that *not applicable* papers were out of scope and to analyze performance on scored papers. Consistent with our previous methods, if both the curator and the classifier agreed regarding the presence or absence of an entity type, then we assumed that the answer was correct and looked no further. Disagreements, in contrast, were classified as false negatives or false positives, with the assumption that the curator is always correct. False negatives occurred when the classifier noted an entity type as missing when it was really present. False positives occurred when the classifier incorrectly noted an entity type as being present when it was missing.

For testing *not applicable* papers (SciScore=0), a curator (NA) went through 232 the previously *not applicable* papers to determine whether each paper was still expected to be scored as a 0 even after the addition of new entity types. From the original 250 papers, 18 (7.2%) papers were removed because they were previously determined to have either no clear methods section (highly theoretical papers, editorials, etc) or contained only supplemental PDFs, which are effectively invisible to our models [14]. Of these 232 papers, 173 (74.6%) were hand scored as 0 and represented papers we expected to still be *not applicable*. We compared each classifier’s output against our curator’s for these 173 papers. A total of 87.9% (152/173) of the papers scored as expected (SciScore=0), and 12.1% (21/173) of the papers contained false positives across the various entity types. Entity types with multiple false positives included attrition (7/21, 33%), randomization (4/21, 19%), field sample permit (3/21, 14%), software tools (3/21, 14%), weight (3/21, 14%), and age (2/21, 10%).

For testing the scored papers, another set containing 250 papers (SciScore >0) was hand curated without exception. Hand-curated data from our first run were supplemented with data for our new criteria, except for statistical tests, which were not tracked (similar to oligonucleotides and plasmids). In all, 2 curators (NA and JM) went through these papers and were blinded to our models’ outputs (50 papers for NA and 200 papers for JM). This information was again compared with our classifiers’ performances; the results of this analysis are shown in Table 2. All entity types had curator-classifier agreement rates >80%; many were >90%. As in our previous analysis, the overall agreement represents the additive probability for instances where multiple resources were mentioned. In all the cases, the agreement rate was measured above the raw classifier *F*_1_ rate.

Overall, there was no significant decline in performance across the criteria featured in either version; any difference in scoring resulted from the addition of new training data or enhanced conditional scoring. As a result of these analyses, we did not seek to further tune the parameters.

**Table 2:** Rates of false negatives, false positives, and overall agreement based on manual analysis of 250 scored papers (SciScore >0) from our data set.

Entity type		False positives	False negatives	Overall agreement
		Size and rate, n (%)	Size and rate, n (%)	Size and rate, (agreed, n) (%)
**Rigor criteria**				
	Institutional review board statement	14 (5.6)	11 (4.4)	225 (90)
	Consent statement	1 (0.4)	11 (4.4)	238 (95.2)
	Institutional animal care and use committee statement	2 (0.8)	17 (6.8)	231 (92.4)
	Field sample permit	19 (7.6)	0 (0)	231 (92.4)
	Euthanasia	6 (2.4)	7 (2.8)	237 (94.8)
	Inclusion and exclusion criteria	10 (4)	17 (6.8)	223 (89.2)
	Attrition	35 (14)	7 (2.8)	208 (83.2)
	Type of replication	0 (0)	3 (1.2)	247 (98.8)
	Number of replications	17 (6.8)	16 (6.4)	217 (86.8)
	General replication	13 (5.2)	16 (6.4)	221 (88.4)
	Randomization of participants into groups	20 (8)	4 (1.6)	226 (90.4)
	Blinding of investigator or analysis	5 (2)	5 (2)	240 (96)
	Power analysis for group size	12 (4.8)	4 (1.6)	234 (93.6)
	Sex as a biological variable	6 (2.4)	21 (8.4)	223 (89.2)
	Age	5 (2)	44 (17.6)	201 (80.4)
	Weight	6 (2.4)	22 (8.8)	222 (88.8)
	Cell line authentication	15 (6)	1 (0.4)	234 (93.6)
	Cell line contamination check	0 (0)	0 (0)	250 (100)
	Protocol identifiers	3 (1.2)	2 (0.8)	245 (98)
	Code availability	4 (1.6)	1 (0.4)	245 (98)
	Code identifiers	0 (0)	2 (0.8)	248 (99.2)
	Data availability	24 (9.6)	0 (0)	226 (90.4)
	Data identifiers	27 (10.8)	3 (1.2)	220 (88)
**Key biological resources**				
	Antibody	2 (0.8)	5 (2)	243 (97.2)
	Organism	3 (1.2)	7 (2.8)	240 (96)
	Cell line	6 (2.4)	4 (1.6)	240 (96)
	Software project and tools	8 (3.2)	38 (15.2)	204 (81.6)

### Text Mining the Open Access Subset of PubMed Central

#### Overview

We downloaded and processed all PubMed Central (PMC; RRID: SCR_004166) articles whose full text was available in the PMC Open Archives Initiative (OAI) data set starting April 2021 (processing took approximately 2 months). The PMC-OAI data set was initially downloaded as multiple directories (1 per journal), containing articles available for text mining. These directories were consolidated into 4 shards (or parts), depending on the number of manuscripts available within each journal. Each shard was then processed using the proposed models. Consistent with our previous RTI study, abstract-only articles and articles without methods sections were excluded [14]. Similarly, articles only available as PDFs were not included within the open access (OA) subset, and, as such, were excluded from our analysis. We included data from journals, institutions, and countries that had published >10 papers per year. This information is available in the Multimedia Appendix 4. We limited our analyses to journals, institutions, and countries that had published >10 papers per category, such as year, if the data were only differentiated by year (eg, all by country vs all by country by year). We obtained data from 2,153,877 manuscripts representing 9398 journals, 37,648 research institutions, and 200 countries (based on research institution metadata in the Research Organization Registry [ROR]).

#### Deduplication and Disambiguation of Research Institutions

We sought to disambiguate the authors’ affiliation strings using the standardized set of institutions listed in the ROR [27]. ROR provides unique identifiers and metadata for many institutions worldwide.

The ROR has developed an application programming interface (API) to search for and retrieve information from its registry. It is able to make a best guess at the institution identifier given an input affiliation string using a combination of substring searches, fuzzy word comparisons, and hard-coded heuristics. Although their API is offered as a web service, initial tests raised concerns of rate-limiting and slow response times for a large volume of requests. However, a developer version was obtained from the ROR [28], which allowed us to run an API instance on a local machine and avoid network concerns. We used the API end point organizations?affiliation= for disambiguating affiliation strings. For each query, a confidence score was provided along with a binary match or no match field. Almost all queries returned a best guess institution from the ROR, although the API did not declare confidence for all queries. We recorded all guesses in our database, whether the API was confident, the confidence using the chosen field of the API response, and the time that the API took on our local machine.

**Figure 1:**
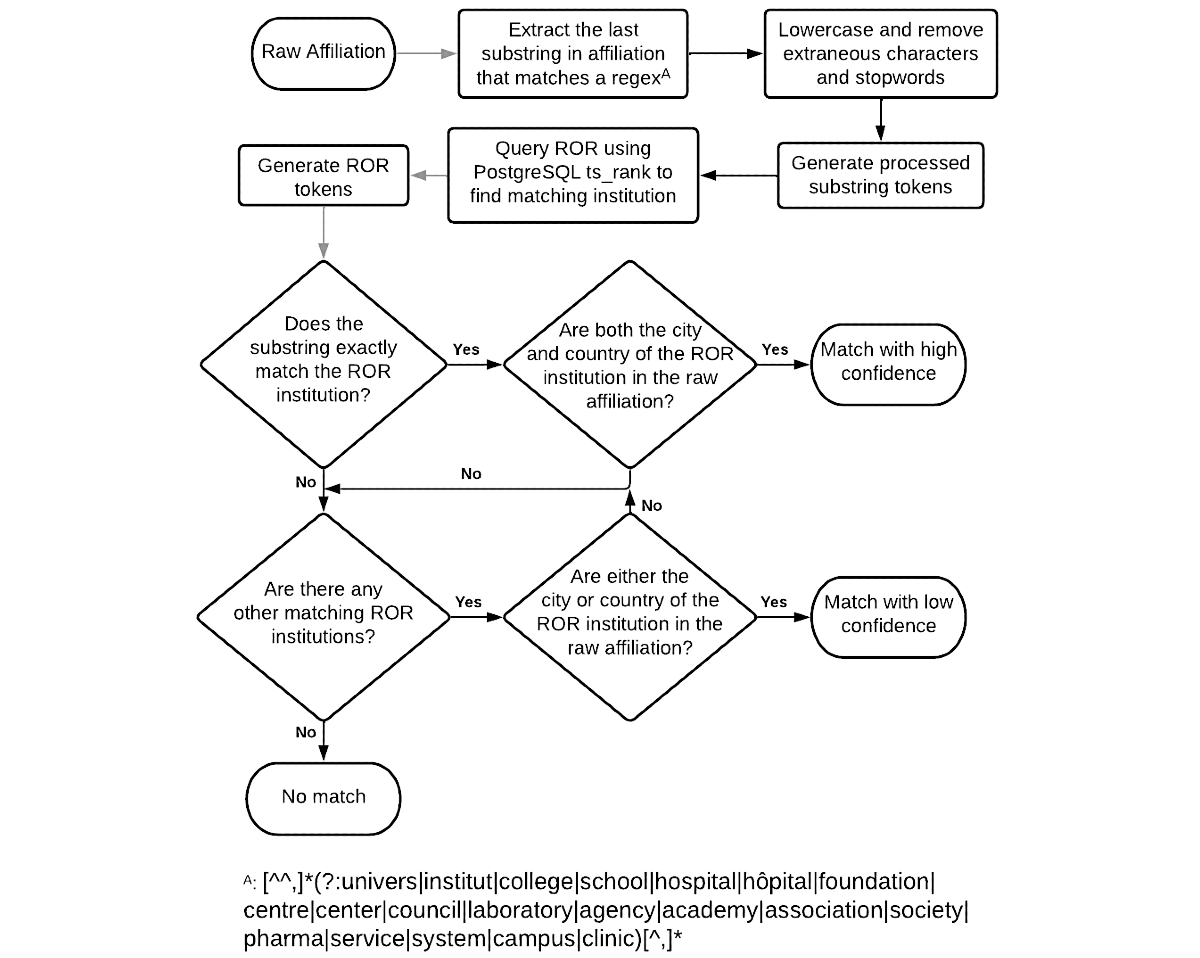
Disambiguation of affiliation strings workflow. ROR: Research Organization Registry; regex^A^: exact regular expression.

We also developed our own tool for disambiguating affiliations (available on GitHub [29]). We used a regular expression (Figure 1) to extract an institution’s name from each affiliation string. Affiliation strings were split on all semicolons, regardless of length, to capture cases in which multiple affiliations were present in a single string. In these cases, a single paper could be included in the counts of multiple institutions (eg, *UCSD*; *UCLA*) where each institution’s paper count would each increase by 1 or be counted multiple times by a single institution (eg, *Department of Computer Science, UCSD; Department of Biological Sciences, UCSD*) where the paper would be included twice in UCSD’s count. ROR data were loaded onto a PostgreSQL instance, and institution names were stored in a *tsvector* column for fast lookup of the regular expression-extracted institution name. The workflow is illustrated in Figure 1.

To compare the performance of our tool with that of ROR’s, 2 curators (JM and PE) matched 200 affiliation strings from a simple random sample of all affiliations from our PMC set (100 per curator) to institutions contained within the ROR database. For cases in which curators could not locate a matching ROR institution, the affiliation string was left blank. A total of 186 strings were matched to ROR institutions. The accuracy was calculated for each tool. Accuracy was defined as the percentage of institutions in which the result of the tool was equal to the result from the hand-curated set. Only when the tool and hand-curated set agreed exactly (ie, either both reported no matching ROR IDs or both reported the exact same ROR ID) was an accurate match declared. Calculations were performed for 2 cases: high confidence matches only and all matches (high and low confidence). The results of this comparison are shown in Table 3.

As shown in Table 3, both algorithms performed similarly in terms of accuracy. Our in-house tool’s speed greatly differentiated itself from ROR’s. As a result of this analysis, we elected to use our in-house tool over ROR’s for institutional disambiguation.

**Table 3:** Affiliation to institution matching: in-house tool compared with the Research Organization Registry (ROR) application programming interface and a human-curated set of 200 affiliations.

Confidence	Time per affiliation (ms)		Accuracy	
	In-house	ROR	In-house	ROR
High only	1.759	400.90	0.5323	0.6666
High and low	9.745	400.90	0.7043	0.7043

#### Department Identification and Grouping

To account for differing reporting standards and expectations across fields, we sought to measure how semantically similar papers are. Specifically, we used abstract similarity measures to group departments of major UK research institutions, so we could compare departments to their analogs at other institutions.

All affiliation strings that contained the strings *United Kingdom*, *Scotland*, *Wales*, or *England* were included. The following regex was used for extracting department names from the affiliation strings:

[^^,]*(?:department|centre|center|section|division| institute|institution|program|school|museum|group)[^,]*

Unwanted characters at the beginning of each affiliation string were removed according to the regex ^[^A-Z]*, and the surrounding whitespace was stripped. All affiliation data along with corresponding PubMed Identifiers were stored in a PostgreSQL table.

For judging semantic similarity across papers, we used the averaged word vectors (normalized by L2) of the abstracts. First, abstracts were extracted from the PMC XML data dumps (all data available before December 12, 2020), excluding articles with a publication type of *Comment*, *Published Erratum*, *Review*, or *Preprint*. Abstract text was stored in the PostgreSQL table along with the PubMed Identifier. Then, a random sample of 1% of all abstracts in the database was used to train fastText [30] word embeddings with default hyperparameters and dimensionality of 300. Then, for each abstract in the table, fastText’s *getSentenceVector* function was used to determine the averaged L2 normalized word vector for each abstract, and the result was stored as a vector in the PostgreSQL table.

To cluster departments based on this similarity measure, we first found the average abstract vector for departments with >200 papers. This was a simple mean of all abstract vectors with an identical department name, previously described, and top-level institution as determined by our in-house disambiguator. Then, using t-SNE as implemented by scikit-learn ([31]; RRID: SCR_002577) with a perplexity of 7, we reduced 300 dimensions to 2 so that similarities between departments could be visualized. Finally, we used scikit-learn k-means clustering on the reduced data to identify 10 clusters of similar departments. To compare the RTIs across departments in each cluster, we found the RTIs across all papers in a given department and ranked departments based on the RTIs within each cluster.

### Statistics

Journals, institutions, and countries were only included in our analyses if more than 10 papers were scored per year unless stated otherwise.

For SciScore named entity classifiers and disambiguation algorithms, we used the standard measures to quantify performance: recall (R), precision (P), and the harmonic mean of R and P (*F*_1_). These values were determined using the following formulas:


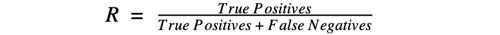
 (1)



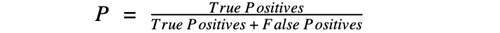
 (2)



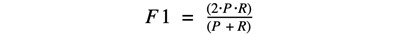
 (3)


False negatives are criteria that were missed by our models but were labeled by a human curator and false positives were incorrectly identified as an entity by our models.

The partial correlation coefficient was calculated using Spearman rank-order correlation coefficient using the following equation:


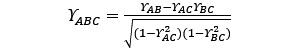
 (4)


where *Y_ABC_* is the correlation between A and B adjusted for C.

### Ethics Approval

We did not obtain institutional review board approval to conduct this study, as we did not use any human or animal participants, thus making this study exempt.

## Results

### Overview

Using our institutional disambiguation model, we obtained data from 2,153,877 articles from 9398 unique journals representing 37,648 institutions across 200 countries. Of these articles, 1,971,824 (91.55%) contained rigor and transparency criteria (SciScore>0; RTI 3.99). The remaining 182,053 (8.45%) articles contained no mention of such criteria (SciScore=0; not applicable). As a result, we did not include these articles in our primary analyses; they did not contain a methods section or were out of scope [14]. We were able to confidently match 1,947,966 articles to 37,067 distinct institutions across 200 countries, where SciScore>0. The RTI data are available in Multimedia Appendix 5.

### Criteria Trends Over Time

**Figure 2:**
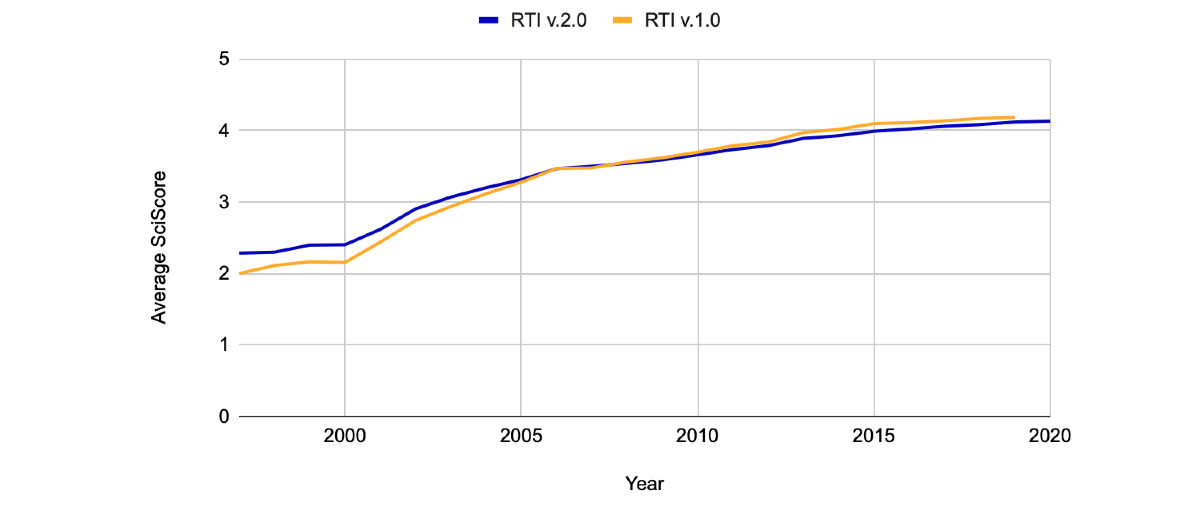
Average score for Rigor and Transparency Index (RTI), version 1.0 (1997-2019) and version 2.0 (1997-2020). PubMed Central- Open Archives Initiative steadily increases over time. Differences between versions are negligible.

**Figure 3:**
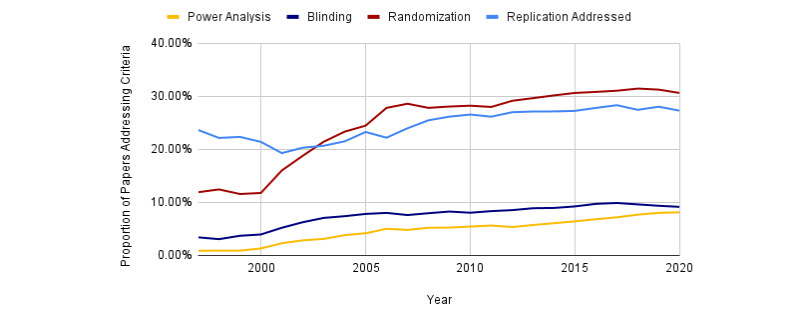
Proportion of papers addressing various bias limiting criteria (ie, blinding, randomization, power, and replication) across all scored papers (1997-2020).

**Figure 4:**
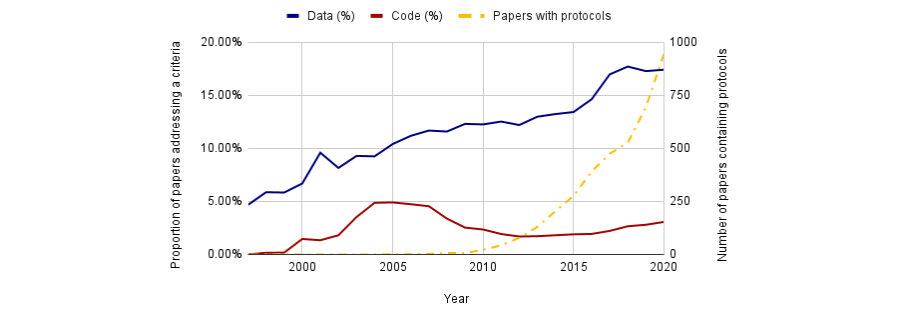
Data, code, and protocol addressment across all papers (1997-2020).

We determined the proportion of papers that addressed individual rigor criteria within the PMC-OAI subset. Data for RTI, version 1.0, represent PMC-OAI manuscripts published between 1997 and 2019. RTI, version 2.0, data are from the PMC-OAI manuscripts published between 1997 and 2020. Both the metrics steadily rise over time, although there is relatively little difference between RTI, version 1.0, and RTI, version 2.0, in terms of their RTIs. As shown in Figure 2, RTI has steadily increased over the last two decades, showing improved levels of transparency within machine-accessible PMC manuscripts. Out of the rigor criteria shown in Figure 3, author addressment of randomization increased the most between 1997 and 2020 (12% to 31%). Blinding (3% to 9%), power analysis (1% to 8%), and replication addressment (24% to 27%), all improved over this timeframe as well. Even at their maximum, blinding and power analysis were addressed in <10% of the studies. Replication addressment represents the percentage of papers that mention replication, number of replications, or type of replication. Figure 4 shows the data, code, and protocol presence across all the papers, regardless of score. Here, we considered a paper to address data presence if the paper had a data availability statement (eg, *all data used within this study is available in the supplementary methods* or *data is available upon request*) or a data identifier (ie, common accession number patterns in data repositories). Code accessibility was determined in a similar manner. We note that this is a conservative estimate of data and code accessibility, as we only checked the methods and materials sections, and some journals place these in a section completely separate from the materials and methods, whereas others use the references section. In addition, we were unable to check if identifiers actually exist owing to slow resolver resolution or if data or code is actually present in the supplementary files. Data addressment (5% to 17%), code addressment (0% to 3%), and the number of protocols cited (0 to 946 papers), all increased between 1997 and 2020.

**Figure 5:**
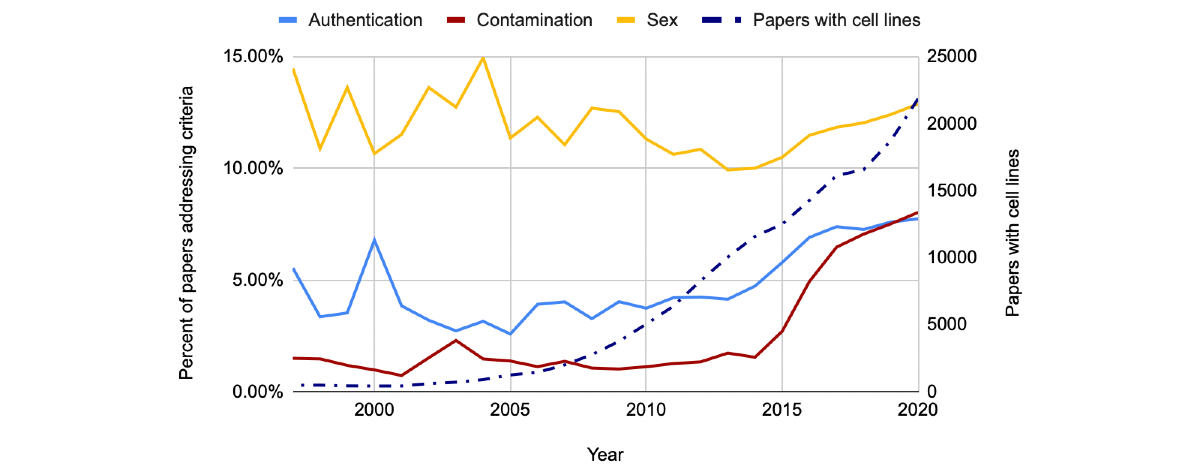
Data shown from 1997 to 2020. Left axis shows the percentage of papers containing cell lines that authenticate, check cells for contamination, and include sex. Right axis shows the number of papers using cell lines each year.

**Figure 6:**
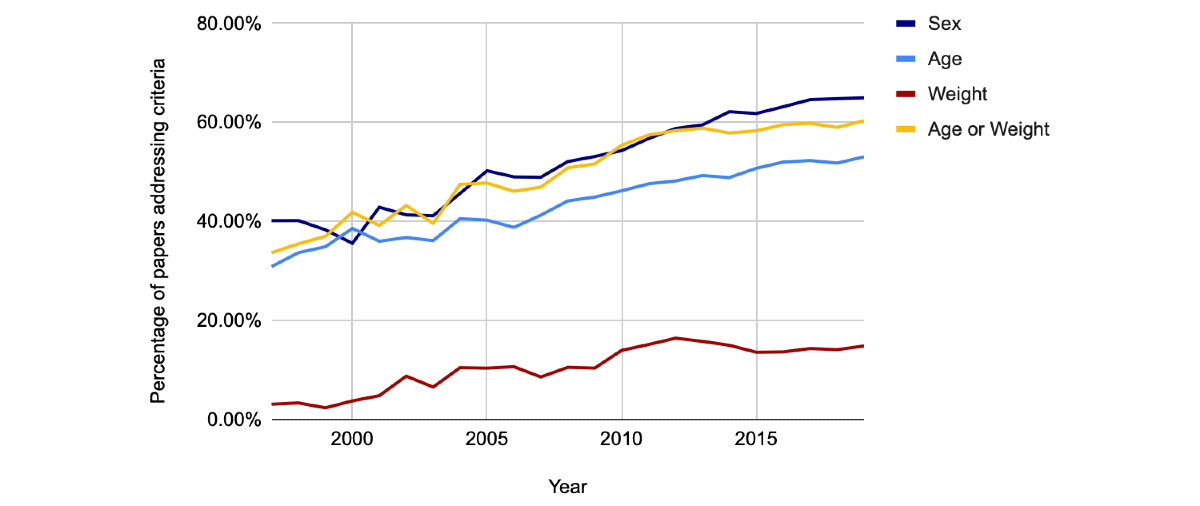
Data from 1997 to 2020. Percentage of papers describing demographic information (sex, age, or weight) that contain at least one transgenic organism.

In Figure 5, when looking at criteria commonly associated with cell line reporting standards (sex, cell line authentication, and contamination), we limited our analysis to papers containing at least one cell line and no IRB or IACUC, as detected by our models. As shown, the number of papers using cell lines continues to grow (470 to 21,854). Within this set, sex did not improve (14% to 13%), whereas the reporting of both cell line authentication (6% to 8%) and contamination (1% to 8%) increased but remained at relatively low levels. As shown in Figure 6, studies containing at least one organism were used to inform our analysis of the organism’s demographic reporting rates. Reporting rates for sex (40% to 65%), age (31% to 54%), and weight (3% to 15%) improved steadily across the board.

### Criteria Across Journals, Research Institutions, and Countries

Among the journals with >10 papers scored in 2020, the top performer in RTI was the *Journal of Neurochemistry* (RTI 6.24). Of the journals with >1000 papers scored in 2020, a total of 2 journals were tied for the lead in RTI, medicine, and nutrients (RTI 5.02). For reference, the RTI across all the papers scored in 2020 was 4.13. Further information on journal performance and journal performance by year is available in Multimedia Appendix 5.

**Figure 7:**
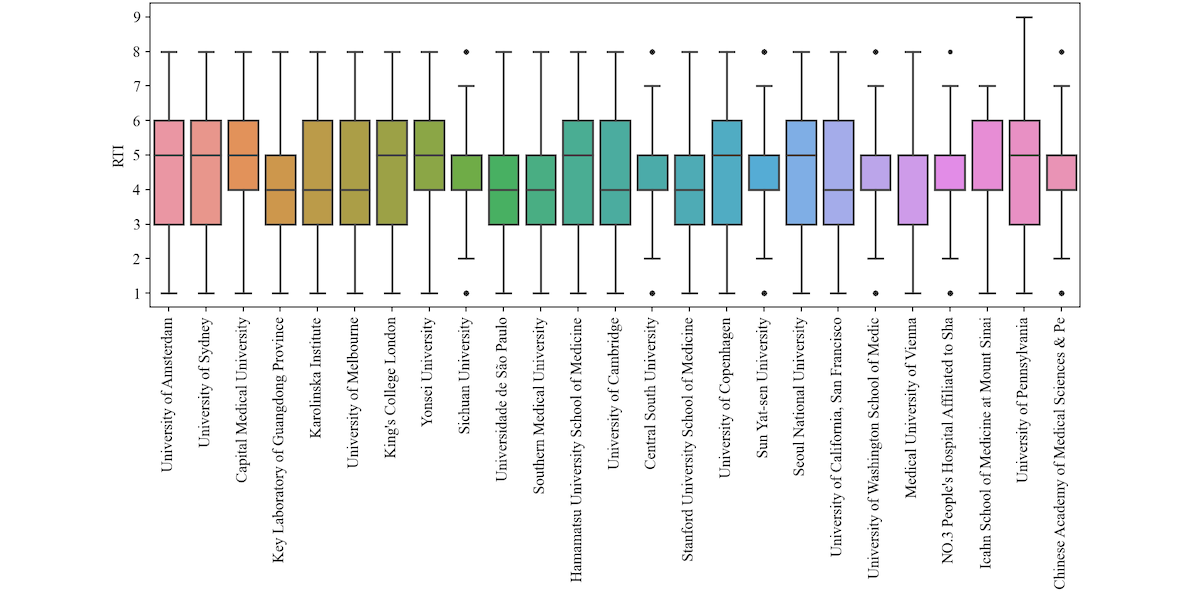
Analysis of Rigor and Transparency Index (RTI) across research institutions in 2020. The left axis represents the RTI. The 50 institutions with the most papers published in 2020 were ranked according to their RTI. The 25 institutions with the highest RTI are shown.

The data in Figure 7 represent 186,045 OA papers published in 2020. The 2 countries with the greatest number of institutions, represented in Figure 7, were China (8/25, 32%) and the United States (5/25, 20%). Many other countries had either 1 or 2 institutions represented. Among individual institutions, Capital Medical University (n=10,125) had the highest RTI (4.75).

We were able to successfully match our institutional data (for institutions with ≥100 papers in 2013) to the names of 110 institutions listed in the data set used by Lepori et al [32] in 2019 to compare university revenues with their publication and citation counts. For the 110 matched institutions, Table 4 shows the correlation calculations between the 3 variables (all from 2013): total number of academic staffs, current total revenue, and RTI. As expected, there was a positive correlation (0.62) between the total number of academic staff and the current total revenue, which makes sense—as staff grows, so do costs. We also performed a partial correlation analysis between the total revenue and RTI, correcting for the total number of academic staffs. This shows that there is a weak negative relationship between an institution’s total revenue and its RTI, although the correlation coefficient (−0.12) suggests that this is not significant. Correlation values were calculated using Spearman rank-order correlation coefficient.

**Table 4:** Spearman rank-order correlation coefficient calculations between the number of academic staffs, the total revenue, and Rigor and Transparency Index (RTI).^a^ The partial correlation coefficient between revenue and RTI was calculated to be -0.1154.

	Total academic staff	Current total revenue	RTI
Total academic staff	1	N/A^b^	N/A
Total current revenue	0.6208	1	N/A
RTI	−0.1209	−0.1648	1

^a^Data from 2013. A partial correlation was calculated between total revenue and RTI correcting for the number of academic staffs.

^b^N/A: not applicable.

### Department Identification and Grouping

**Figure 8:**
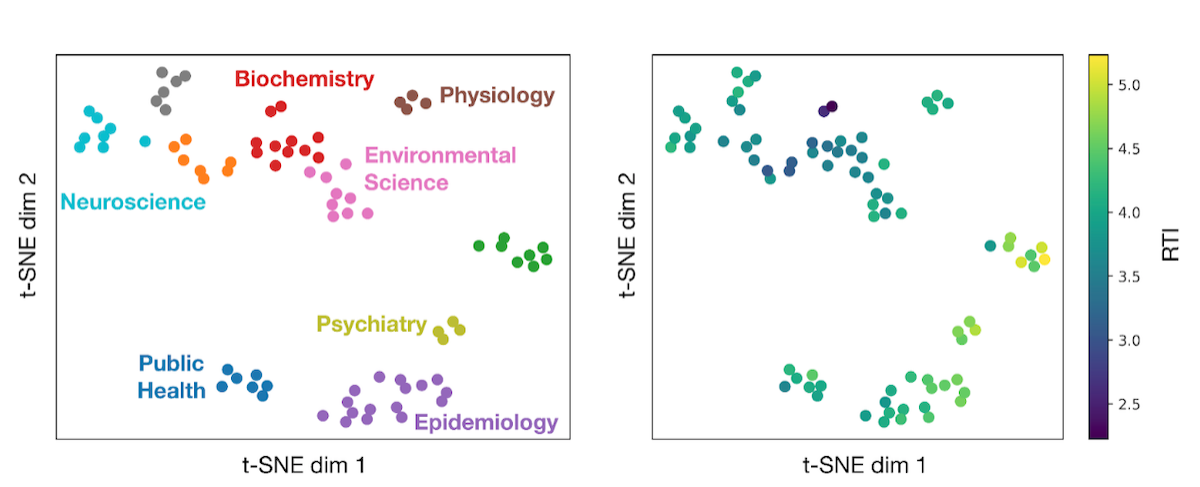
Clustering and Rigor and Transparency (RTI) ranking of the top 80 UK departments by paper count are shown. The t-distributed stochastic neighbor embeddings of the semantic vector representation of each department’s average paper abstract is shown, with k-means clusters indicated by coloring (left panel). Field names are shown for clusters with a single unifying theme among all departments. The labels were added by hand for presentation purposes. We also show the average RTIs of each department (right panel).

Institutional departments should be compared directly to meaningfully compare institutions at more granular levels, as reporting requirements and standards vary across fields. Therefore, we advise against interfield comparisons for this reason. We grouped the largest 80 UK departments by paper count, using the semantic similarities of their abstracts. Following the procedure described in Section 2.2, we computed a t-distributed stochastic neighbor embedding intraplate of abstract vectors across departments and then performed k-means clustering to generate discrete clusters. We visualized each department’s RTI to allow intracluster comparisons (Figure 8). As shown in Figure 8, there are large differences between the RTIs of different fields; for example, the papers of chemistry departments tend to have lower RTIs than psychiatry departments. Therefore, such a clustering is necessary for a fair departmental comparison. We note that departments with alternative spelling are present in this data set, such as the *London School of Hygiene & Tropical Medicine* and *London School of Hygiene [& OR and] Tropical Medicine*. In this analysis, we did not remove these duplicates; however, it is perhaps a good validation that they tended to cluster together and their scores were reasonably similar.

We visualized the RTI for countries with 100 or more scored papers per year available in PMC-OAI between 2010 and 2020 (Multimedia Appendix 6). Each frame represents a different year, where blue represents relatively high scores, and yellow represents relatively low scores. Ethiopia was consistently one of the best performing countries, leading all countries in RTI in 9 out of the 11 years; Ethiopia achieved the highest country average in 2020 (4.98; for reference, RTI in 2020 was 4.13). Norway had the highest RTI papers published in 2010 and 2011. None of the countries consistently had the lowest RTI. The countries with the lowest average in multiple years were Russia (2011, 2013, and 2018), Romania (2012 and 2014), and Ukraine (2015-2017). In terms of volume, the United States and China consistently published the most papers, with the United Kingdom serving as a distant third.

A graphic with coloring scaled to a country’s RTI has been shown over the last 10 years for countries with 100 or more papers. Blue indicates relatively high average values. Yellow indicates relatively low average values. This video is available as an .mp4 file in Multimedia Appendix 6.

### Criteria for Replicating a Study

The Cancer Reproducibility Project, headed by the Center for Open Science and Science Exchange, determined whether the top 50 cancer papers could be reproduced [6]. For each study, the project generated registered reports containing bulleted descriptions of the experimental protocols, data analyses, and replication study reports, which contained free-text descriptions of methods and results from each replicated experiment. The registered reports described their protocols step by step using bullet points, and resources were often only mentioned in reagent tables. Replication studies, in contrast, described both protocols and reagents in paragraphs throughout the methods sections. In addition, the registered reports seemed to focus more on protocol-specific best practices rather than on reporting best practices (eg, RRID use), which makes sense considering that they intend to report the results later. We expect that these differences largely contributed to the differences in scores between the registered reports and the replication studies.

**Figure 9:**
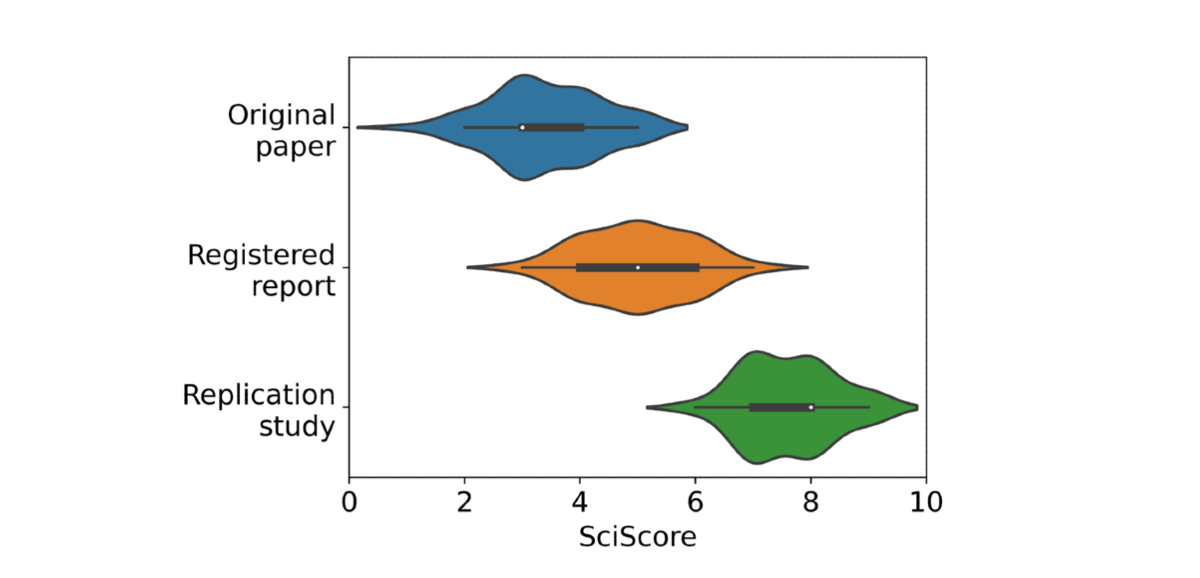
Measured SciScores for Cancer Reproducibility Project papers. Original papers are in blue, registered reports are in orange, and replication studies are in green. A smoothed density plot of scores is shown in solid color. The white dot represents the median score, the thick black line the interquartile range (IQR), and the thin black line 1.5x IQR.

To test our assumption that RTI may serve as a reasonable proxy for replicability, we compared the original studies, which often lacked sufficient detail for performing replication without contacting the original author, with the replicated studies. Figure 9 shows that the replicated reports (RTI 7.61, SD 0.78) were indeed significantly higher (*P*<.001) than their originating reports (RTI 3.39, SD 1.12). The scores of original papers that had responsive authors (RTI 3.45, SD 1.06) and those that did not have responsive authors (RTI 3.33, SD 1.06) were not significantly different on a paired, equal variance *t* test with 1 tail (*P*=.33). The underlying data are provided in Multimedia Appendix 4 [33].

## Discussion

### Principal Findings

In this study, we introduced the latest version of the RTI, that is, RTI, version 2.0, a research reporting metric quantifying research quality and reporting transparency. The RTI lists journals, institutions, and countries with their composite scores and inclusion rates for rigor adherence and resource identifiability. We analyzed a significant number of manuscripts within the OAI subset of PMC, providing an opportunity to see general reporting trends within biomedicine and where we generally fall short within scientific reporting. In addition, we highlight the importance of high-quality reporting and demonstrate RTI’s potential as a replication metric, using manuscripts from the Reproducibility Project: Cancer Biology. As with all generalized metrics, RTI is not perfect, and we do not expect all papers to score a perfect 10. This paper received a score of 7. As with any automated system, we cannot expect to handle all the edge cases. We expect RTI to be generally applicable to biomedical research. Other fields, for example, chemistry and physics, may not fit as well [14]. Although many of these less applicable papers are adequately handled as *not applicable* or through our more general rigor criteria, false positives do occur within automated systems. We are continuously working to improve RTI’s generalizability through additional criteria (eg, data or code availability) and enhanced conditional scoring, where criteria are only factored in when relevant. Our overall aim is not to have every paper score a 10 but rather to help stakeholders improve papers that would otherwise score very poorly.

### Technical Considerations

Unfortunately, the 2 primary limitations present in RTI, version 1.0, are still present in RTI, version 2.0. These issues can be summarized as follows. First, the OA subset represents only a fraction of the total biomedical literature and must therefore be considered a biased subsample. Second, papers with supplementary methods contained in PDFs are still unreadable to our algorithms, resulting in loss of data. We recognize that this is often due to constraints placed on the authors by the journal. As such, we again implore journals to lift restrictions that would limit the impact and reusability of a manuscript. These limitations have been described in our previous work [14].

Owing to the expanded abilities of SciScore, new considerations arose as well. Of these considerations, one of these stemmed from the addition of our data and code resolver, which attempts to resolve identifiers, URLs, and digital object identifiers by checking for their existence in external sources. To process millions of articles in a timely manner, we were forced to place a time restriction on the resolver. If the outside response time was too slow (≥5 seconds), we failed to resolve it, negatively affecting the reliability of our data. Therefore, we will not be able to comment on the validity of the identifiers detected, as we cannot differentiate between a slow outside resource and one that does not exist. In addition, because we only searched the materials and methods sections of the research manuscripts, as defined by Journal Article Tag Suites XML tags, we lost data only mentioned in other sections (eg, results). Anecdotally, this is especially true of criteria such as attrition, which is often mentioned in the results section and code or data availability statements, which can be listed within their own section at the end of manuscripts. We do report these but do not score these items for this reason. We expect to emend these issues in future versions of RTI.

SciScore’s ability to process tables also improved in RTI, version 2.0, which had unintended side effects. Reagents were often counted twice in papers that used reagent tables (eg, STAR [structured, transparent, accessible reporting] methods) in addition to describing the reagents in their methods sections. In an extreme case, Hill et al [34] paper reported using 191 antibodies (listed in their STAR table), but SciScore identified 276 antibodies (identified from both the STAR table and the methods section text). The tool was not able to determine that the antibodies in the text and table were the same reagent for approximately half of the time in this study. This points to the need for continual improvement of artificial intelligence tools, as improvement in some aspects can lead to unintended consequences for others.

### Analysis of Reporting Trends

#### Overview

After failing to replicate key findings in numerous scientific manuscripts, researchers introduced a variety of standards, guidelines, and checklists aimed at improving scientific reporting and with it, scientific reproducibility [10,13,35]. These guides appear to improve scientific reporting to some extent (Figure 2), although this effect seems be context specific [36]. Although researchers should try to ensure that their own manuscripts meet current best practices before submission, enforcing these standards should not fall entirely on journal staff. Researchers increasingly rely on multiple biological or software tools (antibodies, cell lines, plasmids, etc); these tools alone can have extremely complicated best practices, which may not be well understood by all researchers [37-39]. As such, authors, editors, and reviewers, especially in more general topic journals, may struggle to know which best practices to enforce and how to enforce them. In addition, 8% to 9% more papers are produced every year [40], and the current rate is roughly 2 papers added to PubMed every minute. This means that the task of spreading and checking best practices is difficult. Checklists can help guide best practices, and enforcing these checklists should lead to improved reporting standards [41], but given the scale of publishing, the use of automatic checklist tools such as SciScore and others, more focused tools such as Barzooka (continuous data in bar graphs), JetFighter (color-blind accessibility in visualizations), ODDPub (data and code availability), and RipetaScore (authorship, ethics, and data or code availability) [42-45], should help authors and reviewers improve manuscripts and address common checklist items and omissions consistently across many journals. In addition, automatic checklist completion can only help speed up the review process, which is a notoriously slow endeavor [46]. SciScore currently incorporates criteria from sources such as the ARRIVE (animal research: reporting of in vivo experiments) guidelines, the NIH standards, and the Materials Design, Analysis, and Reporting checklist [9,13,35]. Other automated tools check for figure quality or the presence of limitations statements in the discussion section, which is an important part of several checklists. Additional checklist criteria (eg, PRISMA [Preferred Reporting Items for Systematic Reviews and Meta-Analyses]) should be added in future work, but automated tools such as this should be used to improve the reporting quality within the ever-growing literature. The RTI serves as a potential way to track how often these standards are met across a variety of stakeholders at various organizational levels.

#### Trends Across the General Literature

Experimental replication is a technique standardly used across many different fields. Replication metadata are important to report because readers need it to make accurate inferences about the trustworthiness of an experiment [47]. In 2020, Frommlet and Heinze [48] used meta-analysis to analyze 37 mouse experiments published in *Immunity* for experimental replication data. Although we did not replicate their exact study, our results are comparable. We limited our analysis to manuscripts containing a statement addressing IACUC approval and a *Mice* Medical Subject Headings term in 2020. We analyzed a few replication reporting criteria (the proportion of papers containing an explicit replication statement, number of replications, or type of replication). A major difference in our analyses is that Frommlet and Heinze [48] determined the presence of replication when the manuscript contained a figure indicating data representative of multiple experiments or when an explicit statement was made, whereas our classifier was trained exclusively on explicit statements (eg, “experiments were replicated in triplicate”). Of the manuscripts examined by them, 92% (34/37) contained some form of replication, whereas our data showed a far more conservative rate of 44% (1736/3917). In line with our data, Frommlet and Heinze observe that “the exact number [of replications] is frequently not even specified” and “in virtually all cases, [the replication information provided] is insufficient” [48]. Although not directly comparable, our data show that 42% of mouse research papers in 2020 mentioned a number associated with the amount of independent replications and only 6% explicitly mentioned the type of replication they were performing (ie, technical or biological). Although different in specifics, our results both indicate that replication metadata are generally underreported (at least in mice experiments), showing an easy source of potential improvement within research reporting.

Replication is not the only factor that negatively affects research reproducibility. Misidentified and contaminated cell lines continue to be a significant problem, with reported use rates varying between 10% and 50% [49-51]. Some reporting tools such as RRIDs appear to have lessened the incidence rates of problematic cell lines, as researchers are able to more easily look at a specific cell line’s history [26], but there is still more work to be done. The most direct solution is to properly authenticate cell lines in the laboratory. Although different methods are continuing to be developed, short tandem repeat DNA profiling is currently most used [52-54]. However, this process is both time-consuming and expensive [55]. On the basis of our analysis of papers containing at least one cell line from 1997 to 2020, the rates of authentication have increased but are still low (6% to 8%). Similarly, the rate of contamination checks increased from 1% to 8% across the same time frame (Figure 5). In 2015, *Nature* reported that between 2013 and 2015, only 10% of authors submitting cell line–based papers (n=60) reported authenticating their cell lines [56]. The similarity in values indicates that cell line authentication is severely underreported (and most likely underperformed) in a large portion of biomedical literature. *Nature’s* solution was to enhance its current submission policies to require authors to provide further details on cell line testing. This is easier said than done though. In 2010, the *International Journal of Cancer* became the first journal to require cell line authentication information [57]. Overall, this manual effort proved extremely effective, as the number of problematic cell lines published effectively went to 0 after implementation. This came at an administrative cost, as 240 additional hours were required to enforce these guidelines over the course of the 3-year study [58]. Fortunately, much of the work listed (eg, checking the manuscript and cell line–related data entry) can be automated. On the basis of this, we recommend that journals implement stringent cell line authentication requirements similar to those of the *International Journal of Cancer* and make use of automated tools to limit the administrative costs of best practice enforcement. Future studies could compare journal authentication and contamination rates against the specific guidelines implemented by each journal to determine which guidelines and enforcement strategies are most effective. Future models could also differentiate between authentication methods for more granular analysis.

#### Criteria Across Journals, Research Institutions, and Countries

By directly linking institutions with their research manuscripts, we created a way to track and rate an institution’s published output. The latest version of the RTI, that is, RTI, version 2.0, lists an institution's adherence to various reproducibility-related criteria, as well as the identifiability of its research resources (antibodies, organisms, plasmids, etc). The RTI lists the composite scores for multiple entities (ie, journals, institutions, and countries). On the basis of our analysis of the data obtained from the study by Lepori et al [32], there is no strong correlation (*r*=−0.12) between an institution’s RTI and its total revenue, after correcting for the size of the university through the number of academic staffs.

Although indicators such as global rank, funding, and even citations may be, to some extent, richness measures [32,59], the RTI is not. There is no significant correlation, which leads us to believe that research quality is not entirely driven by funding (or how rich a university or country is). Anecdotally, we believe this is largely owing to a researcher’s knowledge of best practices and the community’s ability to implement and enforce them. The first condition may appear to be met as an increasing number of journals implement best practice submission guidelines and checklists, but this is only the first step. These guidelines must be accessible and easily understood if they are to be effectively used [36]. Once the first condition is met, the second condition should follow more easily, especially if aided by automated tooling. We hope that by comparing research institutions based on the quality of their research outputs, they consider rigor and transparency more in their decision-making with the ultimate goal being a shift from *publish or perish* to rigor and reproducibility.

To further encourage this, we aimed to apply RTI comparisons at the departmental level. Different fields can have drastically different reporting requirements and standards, making more granular comparisons far more tenuous. Nominal grouping alone may not be sufficient, as department names may not fully represent the breadth of a department or the nuances of the different subfields within. To mitigate this, we clustered the top 80 UK departments based on the semantic similarity of their abstracts. As shown, the generated clusters aligned remarkably well with department names, despite being fed only semantic abstract information (Figure 8). Not only do these clusters quantitate differences across departments but they also provide new information that cannot be obtained from name alone. For instance, based on other departments within the same cluster, it appears that the Department of Medicine at the University of Oxford focuses on epidemiological or public health research, whereas the Department of Medicine at the University of Cambridge tends to publish cellular biology research. Using our proposed clustering method, we can quantitate such nuanced differences between departments, allowing a like-to-like comparison of RTIs at the departmental level.

After adding both institution- and country-specific data (as well as expanding the entity types detected), we believe that the RTI’s ability to serve as a proxy for good rigor and transparency practices has only been enhanced. Institutions and countries can now more easily identify areas where they fall short in rigor and reproducibility as well as monitor the impact of various scientific policies. We hope that the RTI will continue to highlight the importance of sound scientific practices.

#### Criteria for Replicating a Study

Although we scored >2 million papers across a range of fields, it is difficult to assess whether a particular score has any relevance to the ability of others to replicate a study. Using work done by the Center for Open Science’s Reproducibility Project: Cancer Biology [6], we were able to look at the scores of all papers originally in their study (RTI 3.40, SD 0.95), which researchers used to attempt to replicate the experiments. According to Errington et al [6], none of the original manuscripts contained sufficient detail to attempt to replicate the study, and all required additional information from the authors. To begin replication attempts, Errington et al [6] had to email the original authors and were only able to replicate studies when the original authors responded with additional details. This process is unreliable and slow and results in the loss of a few experiments, as some authors did not respond. Following this, Errington et al [6] generated registered reports, documenting each protocol in a step-by-step manner. After the review, replication reports containing in-depth descriptions of their methods and results were published. These reports were intentionally as rigorous and transparent as possible, sharing all data and codes openly, following resource-specific best practices, and ensuring that all reagents were listed as transparently as possible. As a result, they scored significantly higher (RTI 7.61, SD 0.78) than their originating manuscripts (Figure 9). We assume that these replication papers, where authors paid as much attention to methodological detail as possible, are much more likely to be replicable without additional correspondence. Although we cannot simply describe all *2* papers as not replicable and all *8* papers as replicable, as numerous fields and their subsequent best practices exist, we can state that higher scores are associated with more methodological detail and as such are likely easier to replicate. We encourage biomedicine authors to aim for high scores by ensuring that their methods sections include as much detail as possible.

## Appendix

Multimedia Appendix 1 Criteria detected using SciScore with applicable guideline source, description, and example listed.

Multimedia Appendix 2 Individual Classifier Performance for Named-Entities. Training set size is shown as the number of entities, which represents the total number of entities tagged by our curators as either positive or negative and number of sentences, which represents the total number of sentences containing positive and negative examples as well as some sentences without any entities used in both training and testing.

Multimedia Appendix 3 JSON file containing regular expression patterns used for protocol, data, and code identifiers.

Multimedia Appendix 4 Data underlying figures.

Multimedia Appendix 5 Rigor and Transparency Index.

Multimedia Appendix 6 A graphic with coloring scaled to a country’s Rigor and Transparency Index shown over the last 10 years (2010-2020) for countries with 100 or more papers. Blue shows relatively high averages. Yellow shows relatively low averages.

## Abbreviations

API: application programming interface
ARRIVE: animal research: reporting of in vivo experiments
CRF: conditional random field
NIH: National Institutes of Health
OA: open access
OAI: Open Archives Initiative
PMC: PubMed Central
PRISMA: Preferred Reporting Items for Systematic Reviews and Meta-Analyses
ROR: Research Organization Registry
RRID: research resource identifier
RTI: Rigor and Transparency Index
STAR: structured, transparent, accessible reporting

## Data and Code Availability

The code for retrieving and preprocessing OA subset XML data has been previously published and is open source [60]. SciScore’s source code is not publicly available owing to its proprietary nature. However, the framework and core features of its underlying classifiers have been described in previous studies on Resource Disambiguator for the Web [24]. All Resource Disambiguator for the Web codes are available and may serve as reasonable equivalents to SciScore’s named entity recognition classifier components. The regular expression patterns used for identifier extraction are available in Multimedia Appendix 3 [61]. The code used for institutional disambiguation is available in GitHub [29]. All SQL statements and spreadsheets used in the figures and analyses can be found in Multimedia Appendix 4 [33]. Data sets generated during OA subset *scoring* containing data from individual papers are not publicly available because of their potentially sensitive nature (ie, low scores assigned to published papers may negatively impact scientists producing OA works, significantly more so than researchers publishing closed access works, without giving them the ability to respond to criticism) but are available from the corresponding author upon reasonable request. Summary data for each journal, institution, and country are provided in supplementary files and have been made available in Multimedia Appendix 5 and via SciScore’s RTI webpage [62].
