# Supplementary material for: Establishing Institutional Scores With the Rigor and Transparency Index: Large-scale Analysis of Scientific Reporting Quality
Source: J Med Internet Res. 2022 Jun 27;24(6):e37324. doi: 10.2196/37324 (PMC9274430; doi:10.2196/37324)
Supplement: Multimedia Appendix 1 [file jmir_v24i6e37324_app1.docx]

Table S1: Criteria detected using SciScore with applicable guideline source, description, and example listed

| *Entity Type* | *Source* | *What is this?* |
| --- | --- | --- |
| *Rigor Criteria (5 total points)* | | |
| Institutional Review Board Statement | MDAR | A statement (usually a single sentence) addressing IRB approval for biomedical research involving human subjects (or why IRB approval was not required). |
| *Example*: All human work was conducted under human subjects protocols approved by the Stanford Institutional Review Board (IRB), the University of Michigan UM-IRBMED, and the Ethical Committee of d’Ile de France II.  *Example:* The trial was approved by the NRES Committee London—South East. | | |
| Consent Statement | MDAR | A statement (usually a single sentence) addressing subject/patient consent in human research (or why consent was not required). |
| *Example*: Written informed consent was obtained from parents of all participating children and oral assent was obtained from 7-year olds.  *Example:* All infants were enrolled with informed parental permission under a protocol that was reviewed and approved by the Institutional Review Boards of the respective study sites. | | |
| Institutional Animal Care and Use Committee Statement | MDAR, ARRIVE | A statement (usually a single sentence) addressing IACUC ethical approval for research involving vertebrate organisms. |
| *Example*: All animal experiments were performed in accordance with relevant guidelines and regulations and were approved by the University of Pennsylvania Institutional Animal Care and Use Committee (IACUC).  *Example*: All animals used in this study were treated in accordance with UK Animal (Scientific Procedures) legislation and under the appropriate project licenses, national and local ethical approval. | | |
| Field Sample Permit ^a^ | MDAR | A statement disclosing the relevant permits obtained (including the name of the permitting authority) for field studies (or why approval was not required). |
| *Example*: Permission to conduct field surveys on each location was given by the individual landowners concerned, and by the regulatory authority (Natural England) in those situations where the field site was afforded protected status (i.e. Site of Special Scientific Interest). | | |
| Euthanasia ^a,b^ | AVMA Guidelines | The mention of culling or euthanasia for the animals used in a research experiment. |
| *Example*: Mice injected with CGG-NP23 were boosted on day 21 with the same inoculum and killed on day 28. | | |
| Euthanasia Agent ^a,b^ | AVMA Guidelines | The mention of an agent or method (i.e. cervical dislocation or carbon dioxide inhalation) used in the euthanasia of research animals. |
| *Example*: Twelve hours after the final doses, the animals were euthanized by cervical dislocation. | | |
| Inclusion & Exclusion Criteria ^a^ | Landis et al., 2013 (NIH), MDAR, CONSORT, ARRIVE, PRISMA | A statement or statements reporting the criteria prospective subjects must have (or must not have) in order to be included/excluded in a study. |
| *Example*: Exclusion criteria were pregnancy, severe medical conditions, abnormal laboratory baseline values, unstable psychiatric features (e.g., suicidal), a history of alcoholism or drug abuse, epilepsy, brain trauma with loss of consciousness, neurological illness, or a concomitant Axis I psychiatric disorder. | | |
| Attrition ^a^ | Landis et al., 2013 (NIH), MDAR, ARRIVE | A sentence reporting whether any sample or data point was omitted (participant drop out or intentionally excluded by author). This includes sentences that report no attrition. |
| *Example*: One participant withdrew from the yoga exercise group due to personal consideration. | | |
| General Replication ^a^ | Landis et al., 2013 (NIH), MDAR | The mention of experimental replication or repetitions within an experiment. |
| *Example*: The experiment was replicated four times. | | |
| Type of Replication ^a^ | Landis et al., 2013 (NIH), MDAR | A description of the type of replication being performed (e.g. technical replicant or biological replicant - biologically distinct samples or repeated measures of the same sample). |
| *Example*: Each real-time PCR experiment included technical replicates, in a final volume of 15 µL. | | |
| Number of Replications ^a^ | Landis et al., 2013 (NIH), MDAR | A brief mention of the number of times an experiment was independently performed. |
| *Example*: The experiment was replicated four times. | | |
| Randomization of subjects into groups | Landis et al., 2013 (NIH), MDAR, CONSORT, ARRIVE | Considered addressed when a statement describing whether randomization was used (e.g. assigning subjects to experimental groups, positions in a multiwell device, processing order, etc.). |
| *Example*: Animals were assigned to experimental groups using simple randomization.  *Example*: Communication with schools, and elicitation of willingness to participate, was conducted before the village-level randomization took place. | | |
| Blinding of investigator or analysis | Landis et al., 2013 (NIH), MDAR, CONSORT, ARRIVE | A statement discussing the degree to which experimenters were unaware (or blinded) of group assignment and/or outcome assessment. |
| *Example*: Responses were then scored by an experimenter blinded to injection condition and experimental cohort.  *Example*: All the analysis was performed by a person unaware of the experimental question. | | |
| Power analysis for group size | Landis et al., 2013 (NIH), MDAR, CONSORT, ARRIVE | A statement addressing how (and if) an appropriate sample size was computed. |
| *Example*: Sample size was based on estimations by power analysis with a level of significance of 0.05 and a power of 0.9.  *Example:* Sample size calculation was done for the primary aim of this study, i.e. FMD, as reported previously. | | |
| Sex as a biological variable | MDAR, NIH, CONSORT, ARRIVE | Reporting the sex of any and all organisms, cell lines, and human subjects. |
| *Example*: Six healthy adult rhesus macaques (Macaca mulatta) of Chinese origin (4–8 kg, three males and three females, 4–8 years old) were inoculated intramuscularly (i.m.) with 1,000 pfu of EBOV Makona strain.  *Example:* In each session, the behavior of each mother was recorded every 2 min. | | |
| Age ^a^ | CONSORT, ARRIVE | A statement reporting the age (or stage of life) of an experimental subject or organism. |
| *Example*: All mice were 8–16 weeks of age. | | |
| Weight ^a^ | ARRIVE | A statement reporting the weight of an experimental organism. |
| *Example*: One Thoroughbred healthy adult horse (540 kg body mass) from the Royal Veterinary College (RVC) participated in the study. | | |
| Cell Line Authentication | MDAR, NIH | A statement detailing how the cell lines used were authenticated (e.g. short tandem repeat analysis). This is only required when cell lines are detected. |
| *Example*: MOLM-14 cells were authenticated by STR profiling and flow cytometry.  *Example:* All cell lines were obtained from ATCC, tested negative for mycoplasma, and their identity was verified by short tandem repeat analysis (Promega GenePrint 10 System). | | |
| Cell Line Contamination Check | MDAR, NIH | A statement addressing the mycoplasma contamination status of the cell lines used. This is only required when cell lines are detected. |
| *Example*: All cell lines were obtained from ATCC and tested negative for mycoplasma contamination.  *Example*: All cell lines were confirmed to be mycoplasma free using a PCR-based detection strategy with positive and negative controls. | | |
| Protocol Identifiers ^a^ | MDAR, CONSORT (clinical trial number required), PRISMA | We use a series of regular expressions to find and link certain patterns (usually accession numbers) with their corresponding database. Protocol identifiers include registered clinical trials (clinicaltrials.gov and EU Clinical Trials Register) and protocol repositories (protocols.io and protocol exchange). |
| *Example*: To study the effect of dutasteride on Abi metabolism, serum samples were collected from patients treated on a phase II clinical trial at Dana-Farber Cancer Institute (NCT01393730). | | |
| Code Availability ^a^ | MDAR | A sentence disclosing the availability of any computer code (either newly generated or previously created) that is essential for replicating the main findings of the study. |
| *Example*: Image analysis was performed with ImageJ software macro (code available upon request). | | |
| Code Identifiers ^a^ | MDAR | We use a series of regular expressions to find and link certain patterns (usually accession numbers or URLs) with their corresponding code repositories. |
| *Example*: All scripts used for the analyses in this paper are available at the Github repository (https://github.com/vplagnol/recursive_splicing). | | |
| Data Availability ^a^ | NIH, MDAR, ARRIVE | A sentence disclosing the availability of any data (either newly generated or from a previous study) that is essential for replicating the main findings of the study. |
| *Example*: All other relevant data that support the conclusions of the study are available from the authors on request. | | |
| Data Identifiers ^a^ | MDAR | We use a series of regular expressions to find and link certain patterns (usually accession numbers) with their corresponding data repositories. |
| *Example*: The complete results are uploaded in NCBI GEO as GSE75387. | | |
| *Key Biological Resources (5 total points)* | | |
| Antibody | MDAR, NIH, STAR, RRID | The models attempt to find all antibody entities within the methods section. “Identifiable” antibodies are reported with any metadata required to uniquely identify the antibody used such as vendor, catalog number, clone ID, batch number, or RRID. |
| *Example*: ATF3 antibody (Santa Cruz Biotechnology) was used at 1:2000.  Example: Slices were then washed (3x) and placed in PBS containing the following; 1% (vol/vol) normal goat serum, 1% (vol/vol) BSA, 0.25% (vol/vol) Triton X-100, and mouse monoclonal anti-5.8S rRNA, clone Y10b at 1:500 (Abcam, ab37144, RRID: [AB_777714](https://scicrunch.org/resolver/AB_777714)) overnight at 4°C. | | |
| Organism | MDAR, NIH, RRID, STAR, ARRIVE | The models attempt to find all organism entities within the methods section. “Identifiable” organisms are reported with any metadata required to uniquely identify the organism used such as vendor, catalog number, or RRID. |
| *Example (mouse)*: Adult (10-12 weeks; 25-30g) male C57BL/6 and TH-Cre mice were group-housed until surgery.  *Example (fly)*: To generate PIP821bpΔ the following sgRNA was generated 5’-GCAGGAGGAGGTACAGCGGG-3’ and cloned into pU6-2-BbsI-gRNA (DGRC #1363) and then subsequently injected into w1118; vas-Cas9 (RRID:BDSC_51324, Rainbow Transgenics).  *Example (fish):* The transgenic lines used in this study were Tg(kdrl:EGFP)s843 (Jin et al., 2005), Tg(lyve1b:DsRed2)nz101, Tg(lyve1b:EGFP)nz150 (Okuda et al., 2012), Tg(mpeg1:EGFP)gl22, Tg(mpeg1:Gal4FF) gl25 (Ellett et al., 2011), Tg(lyz:EGFP)nz117 (Hall et al., 2007), Tg(i-fabp:RFP)as200 (Her et al., 2004), Tg(UAS-E1b:nfsB-mCherry)c264 (Davison et al., 2007) and Tg(-8.mpx:KalTA4)gl28. | | |
| Cell Line | MDAR, NIH, STAR, RRID | The models attempt to find all cell line entities within the methods section. “Identifiable” cell lines are reported with any metadata required to uniquely identify the cell line used such as vendor, catalog number, or RRID. |
| *Example*: The lung cancer cell line, H1299, was obtained from the American Tissue Culture Collection (Manassas, VA).  *Example:* J774A.1 murine monocytes and macrophages (ATCC, number TIB-67) were cultured at 37 °C in a humidified air/carbon dioxide (CO2) (19:1) atmosphere in RPMI medium supplemented with 10% (v/v) heat-inactivated fetal bovine serum, penicillin (100 IU/mL), streptomycin (100 µg/mL), and amphotericin B (250 ng/mL). | | |
| Plasmid | STAR, RRID | The models attempt to find all plasmid entities within the methods section. Plasmids were not used in this analysis. |
| *Example*: The constructions were prepared using the vector *pSpCas9(BB)-2A-Puro (PX459) V2.0*, which was a gift from Feng Zhang (Addgene plasmid #62988; RRID: Addgene_62988).  *Example:* For expression in HEK293 cells, INF2 was first subcloned into pGADT7.3 (BspEI/XmaI-XhoI) and then into pEGFP-C3 (EcoRI-SalI). | | |
| Oligonucleotide ^c^ | STAR, MDAR | The models attempt to find all oligonucleotide entities within the methods section. Oligonucleotides do not impact score and were not used in this analysis. |
| *Example*: Activating Notch1 mutations in mouse models of T-ALL, Blood 2006 107:781–785), including one new oligonucleotide primer pair: Ex34B-f: 5′-GCCAGTACAACCCACTACGG-3′; Ex34B-r: 5′-CCTGAAGCACTGGAA-AGGAC-3′.  *Example*: Primers used were GRHL2-1-424-F (TATATAGGATCCATGTCACAAGAGTCGGACAA), GRHL2-1-424-R (ATATAAAGATCTT­TTTCTTTCTGCTCCTTTGT), GRHL2-438-625-F (TAAATTAGATCTAAAGGCCAGGCCTCCCAA­AC), and GRHL2-438-625-R (TTATATGTCGACCTAGATTTCCATGAGCGTGA). | | |
| Software Project/Tool | STAR, RRID, PRISMA | The models attempt to find all software tools within the methods section. “Identifiable” tools are reported with an RRID or are able to be uniquely identified through a distinct name/URL. |
| *Example*: Image J was used to process and analyze raw images (Extended Data Video 2, and 3).  *Example:* All simulations were performed using the NEURON simulation environment (Carnevale and Hines, 2006 ). | | |
| Statistical Tests ^a,c^ | MDAR, CONSORT, ARRIVE | A statement reporting the statistical tests used during the experiment, ideally including a justification (i.e. whether the tests’ assumptions are met). |
| *Example:* Kruskal-Wallis test was used to compare three or more groups. | | |
| ^a^ RTI v.2.0; ^b^ Grouped together in RTI v.2.0, ^c^ Not captured in RTI v.2.0 | | |
