# Supplementary material for: Establishing Institutional Scores With the Rigor and Transparency Index: Large-scale Analysis of Scientific Reporting Quality
Source: J Med Internet Res. 2022 Jun 27;24(6):e37324. doi: 10.2196/37324 (PMC9274430; doi:10.2196/37324)
Supplement: Multimedia Appendix 2 [file jmir_v24i6e37324_app2.docx]

Table S2: Individual Classifier Performance for Named-Entities. Training set size is shown as the # of entities, which represents the total number of entities tagged by our curators as either positive or negative and # of sentences, which represents the total number of sentences containing positive and negative examples as well as some sentences without any entities used in both training and testing.

| *Entity Type* | *F1* | *Precision* | *Recall* | *Training Set Size* (# of entities/# of sentences) |
| --- | --- | --- | --- | --- |
|  | Mean ± SD | Mean ± SD | Mean ± SD |  |
| *Rigor Criteria (5 total points)* | | | | |
| Institutional Review Board Statement | 81.41 ± 3.62 | 84.45 ± 5.26 | 79.57 ± 8.83 | 340/78,170 |
| Consent Statement | 94.75 ± 1.68 | 96.29 ± 2.42 | 93.38 ± 3.63 | 373/78,170 |
| Institutional Animal Care and Use Committee Statement | 81.30 ± 4.20 | 89.30 ± 4.60 | 74.89 ± 6.12 | 591/78,170 |
| Field Sample Permit ^a^ | 62.14 ± 6.58 | 59.54 ± 11.18 | 67.09 ± 8.01 | 20/69,042 |
| Euthanasia ^a,b^ | 98.39 ± 0.57 | 98.48 ± 0.83 | 98.27 ± 0.85 | 22/69,042 |
| Euthanasia Agent ^a,b^ | 86.41 ± 3.35 | 90.22 ± 1.87 | 83.04 ± 5.35 | 102/69,042 |
| Inclusion and Exclusion Criteria ^a^ | 88.22 ± 1.17 | 85.68 ± 2.33 | 91.01 ± 1.86 | 137/69,042 |
| Attrition ^a^ | 40.05 ± 5.06 | 50.34 ± 6.84 | 33.62 ± 5.29 | 308/69,042 |
| Type of Replication ^a^ | 97.71 ± 1.99 | 97.50 ± 3.14 | 98.01 ± 2.90 | 6/69,042 |
| Number of Replications ^a^ | 79.10 ± 2.35 | 79.54 ± 2.86 | 78.73 ± 2.51 | 115/69,042 |
| General Replication ^a^ | 92.09 ± 1.97 | 92.03 ± 1.78 | 92.18 ± 2.73 | 49/69,042 |
| Randomization of subjects into groups ^c^ | 83.05 ± 3.04 | 80.25 ± 5.05 | 86.45 ± 4.64 | 368/52,945 |
| Blinding of investigator or analysis ^c^ | 78.96 ± 12.38 | 77.74 ± 17.16 | 81.79 ± 10.32 | 183/52,945 |
| Power analysis for group size ^c^ | 64.45 ± 29.37 | 73.74 ± 34.13 | 59.50 ± 26.91 | 81/52,945 |
| Sex as a biological variable | 88.32 ± 3.91 | 87.94 ± 6.03 | 88.93 ± 3.52 | 862/52,945 |
| Age ^a^ | 85.21 ± 1.87 | 88.58 ± 3.28 | 82.23 ± 2.83 | 1271/69,042 |
| Weight ^a^ | 80.29 ± 4.56 | 89.22 ± 5.99 | 73.68 ± 8.07 | 338/69,042 |
| Cell Line Authentication ^c^ | 54.08 ± 11.88 | 85.70 ± 10.78 | 41.15 ± 12.82 | 155/14,792 |
| Cell Line Contamination Check ^c^ | 91.70 ± 5.24 | 93.35 ± 7.15 | 90.65 ± 7.05 | 151/14,792 |
| Protocol Identifiers ^a^ | 17 patterns for 12 datasources (clinical trials (US & EU), protocol exchange, STAR protocols, JOVE, Bio-protocol, MethodsX, nature protocols, springer protocols, biotechniques, PROSPERO, protocols.io) | | | |
| Code Availability ^a^ | 90.24 ± 1.90 | 91.77 ± 3.30 | 88.92 ± 3.01 | 10/69,042 |
| Code Identifiers ^a^ | 4 patterns for 4 datasources (github, google code, sourceforge, bitbucket) | | | |
| Data Availability ^a^ | 82.43 ± 3.51 | 83.06 ± 5.75 | 82.06 ± 3.52 | 15/69,042 |
| Data Identifiers ^a^ | 28 patterns for 18 datasources (doi, genomeRNAi, GEA, dbGAP, dbSNP, GEO, SRA arrayexpress, JGA, EGA, metabolights, peptide atlas, proteomeXchange, Flow repository, Biostudies, ClinVar, MassivE, pcddb) | | | |
| *Key Biological Resources (5 total points)* | | | | |
| Antibody | 78.94 ± 2.62 | 86.89 ± 3.78 | 72.46 ± 3.20 | 16,772/53,216 |
| Organism | 66.05 ± 4.70 | 79.91 ± 6.28 | 56.64 ± 5.75 | 4,439/45,500 |
| Cell Line | 70.07 ± 5.95 | 86.48 ± 3.27 | 59.34 ± 8.03 | 1,763/45,500 |
| Plasmid | 79.62 ± 3.35 | 92.53 ± 3.80 | 70.09 ± 4.85 | 2,568/63,400 |
| Oligonucleotide ^d^ | 83.03 ± 9.05 | 95.28 ± 3.13 | 74.94 ± 13.90 | 1,893/63,400 |
| Software Project/Tool | 89.03 ± 0.90 | 92.49 ± 2.08 | 85.84 ± 1.10 | 10,161/19,002 |
| Statistical Tests ^a,d^ | 96.96 ± 0.43 | 96.81 ± 0.83 | 97.11 ± 0.97 | 1,360/69,042 |
| ^a^ RTI v.2.0; ^b^ Grouped together in RTI v.2.0, ^c^ Performance from RTI v.1.0, ^d^ Not captured in RTI v.2.0 | | | | |
